# Whole-Genome Sequence of *Streptococcus suis* Serotype 4 Reference Strain 6407

**DOI:** 10.1128/genomeA.00770-14

**Published:** 2014-08-14

**Authors:** Kaicheng Wang, Jiming Chen, Huochun Yao, Chengping Lu

**Affiliations:** a China Animal Health and Epidemiology Center, Qingdao, China; b College of Veterinary Medicine, Nanjing Agricultural University, Nanjing, China

## Abstract

We report here the second complete genome sequence of *Streptococcus suis* serotype 4 (strain 6407). The genome is 2,292,360 bp in length, covering 2,239 coding sequences, 58 tRNAs, and 4 rRNA loci.

## GENOME ANNOUNCEMENT

*Streptococcus suis* is one of the most important pathogens in the porcine industry and is responsible for causing septicemia, meningitis, and many other infections. In addition, it is an emerging zoonotic agent responsible for septicemia with or without septic shock, meningitis and other less common infections in humans (1). During the last decade, the number of *S. suis* infections reported worldwide has increased significantly, with most cases reported in Asia (2).

A total of 33 serotypes of *S. suis* have been described and are defined based on the antigenicity of their capsular polysaccharide (2). Among them, serotypes 1, 2, 4, 5, 14, 16, 21, and 24 have caused human infections (3–8). Strains isolated from diseased pigs primarily belong to serotype 2 in most countries, followed by serotypes 3, 4, 5, 7, 8, and 1/2 in Asian countries (9–11), and serotypes 3, 1/2, 4, 7, and 8 in Canada (12). To date, genome sequences of *S. suis* have not been reported except for serotypes 1, 2, 3, 7, 9, 14 16, and 1/2 (13–15). Although *S. suis* serotype 4 is zoonotic and rather prevalent worldwide, its genome has not been reported. To facilitate studies, we sequenced a reference strain of *S. suis* serotype 4. The strain, 6407, was isolated from a clinical diseased pig in Denmark (16), kindly obtained from M. Gottschalk (Faculté de Médicine Vétérinaire, Université de Montréal, Saint-Hyacinthe, Quebec, Canada).

The complete genome sequence was determined by an Illumina Miseq system. Assembly was performed using SOAPdenovo. Gaps were filled by primer walking and sequencing of PCR products. The assembly of the genome was further verified by PCR. Coding sequences (CDSs) were predicted using Glimmer 3.02 and GeneMarkS (17), and further examined with the nonredundant protein database through BLASTp. tRNAs and rRNAs were identified using tRNAscan-SE and RNAmmer (18), respectively.

The genome of 6407 was found to have an orientation similar to the majority of the published genomes of *S. suis* in GenBank (13). The genome of strain 6407 consists of a single circular chromosome which is 2,292,360 bp in length, with a G+C content of 40.98%. A total of 2,239 CDSs that account for 97.4% of the genome, 58 tRNAs, and 4 rRNA loci were identified in the 6407 genome.

The function of the CDSs is mainly related to carbohydrate transport and metabolism, translation, ribosomal structure, and biogenesis. There are 4 phage-related regions and 7 prophage elements in the 6407 genomes. The genome of 6407 harbors some virulence associated genes (19), including *epf*, *ssp*A, *srt*A, *pgd*A, *gtf*A, and *fbps*. Some other virulence associated genes, *mrp*, *ofs*, *zur*, *dlt*A, *gln*A, and *sly*, were absent in the 6407 genome.

### Nucleotide sequence accession number.

The complete genome sequence of *S. suis* serotype 4 (strain 6407) has been assigned GenBank accession number CP008921.
